# Procedural times with robotic-assisted bronchoscopy: a high volume single-center study

**DOI:** 10.1177/17534666241277668

**Published:** 2024-09-05

**Authors:** Kim Styrvoky, Audra Schwalk, David Pham, Kristine Madsen, Hsienchang Chiu, Muhanned Abu-Hijleh

**Affiliations:** Department of Medicine, Division of Pulmonary and Critical Care Medicine, University of Texas Southwestern Medical Center, 5323 Harry Hines Boulevard, Dallas, TX 75390, USA; Department of Medicine, Division of Pulmonary and Critical Care Medicine, University of Texas Southwestern Medical Center, Dallas, TX, USA; Department of Medicine, Division of Pulmonary and Critical Care Medicine, University of Texas Southwestern Medical Center, Dallas, TX, USA; Department of Medicine, University of Texas Southwestern Medical Center, Dallas, TX, USA; Department of Medicine, Division of Pulmonary and Critical Care Medicine, University of Texas Southwestern Medical Center, Dallas, TX, USA; Department of Medicine, Division of Pulmonary and Critical Care Medicine, University of Texas Southwestern Medical Center, Dallas, TX, USA

**Keywords:** cone beam computed tomography, lung nodule, procedure scheduling, radial endobronchial ultrasound, robotic bronchoscopy

## Abstract

**Background:**

Incidental and screen-detected pulmonary nodules are common. The increasing capabilities of advanced diagnostic bronchoscopy will increase bronchoscopists’ procedural volume necessitating optimization of procedural scheduling and workflow.

**Objectives:**

The objectives of this study were to determine total time in the procedure room, total bronchoscopy procedure time, and robotic-assisted bronchoscopy procedure time longitudinally and per specific procedure performed.

**Design:**

A single-center observational study of all consecutive patients undergoing shape-sensing robotic-assisted bronchoscopy (RAB) biopsy procedures for the evaluation of pulmonary lesions with variable probability for malignancy.

**Methods:**

Chart review to collect patient demographics, lesion characteristics, and procedural specifics. Descriptive and comparative statistics are reported.

**Results:**

Actual bronchoscopy procedure time may decrease with increased institutional experience over time, however, there is limited ability to reduce non-bronchoscopy related time within the procedure room. The use of cone beam computed tomography (CBCT), rapid on-site evaluation (ROSE), and performance of staging endobronchial ultrasound transbronchial needle aspiration (EBUS-TBNA) in a single procedure are each associated with additional time requirements.

**Conclusion:**

Institutional procedural block times should adapt to the nature of advanced diagnostic bronchoscopy procedures to allow for the accommodation of new modalities such as RAB combined with other technologies including radial endobronchial ultrasound, CBCT, ROSE, and staging linear EBUS. Identifying institutional median procedural times may assist in scheduling and ideal block time utilization.

## Plain language summary

Times necessary to perform robotic assisted bronchoscopy biopsy procedures at a single hospital

Background: Lung lesions and nodules are commonly seen on computed tomography (CT) scans. With advances in technology, more of these lesions are being biopsied with robotic assisted bronchoscopy (RAB) procedures, leading to increased demand. Health care providers who perform these procedures have finite available time in which they must accommodate all their procedures. Understanding procedure times is necessary to fully utilize schedules. Methods and aims overview: We describe our experience of 5 pulmonologists performing 700 robotic assisted bronchoscopies at a single hospital. Our aim is to describe the time needed for the robotic bronchoscopies over time and with specific procedures. Results and conclusion: We find that as more robotic assisted bronchoscopies are performed, the overall procedure time may decrease. Using cone beam computed tomography during the procedure, having on- site pathology review of biopsies, and obtaining biopsies of lymph nodes may lengthen the procedure time. The time spent preparing the patient for the procedure excluding the bronchoscopy remained stable. Understanding the time necessary based on what is performed during the procedure will allow it to be scheduled for the appropriate amount of time. As a result, procedure days can be fully optimized, minimizing scheduling impacts on patients and health care workers.

## Introduction

Lung nodules are a common radiographic finding seen in an estimated 11%–33% of chest imaging with at least 1.5–2 million new nodules detected annually in the United States.^1^ The identification and evaluation of lung nodules will increase secondary to multiple factors: (1) implementation of screening programs, (2) increasing awareness of lung cancer prevalence and risk factors, (3) high utilization of chest imaging for other diagnostic purposes. Current guidelines recommend non-surgical tissue biopsy via percutaneous or guided bronchoscopy approaches for patients with intermediate to high-risk probability for malignancy. Compared to percutaneous computed tomography (CT) guided sampling, advanced diagnostic bronchoscopy may also encompass various diagnostic and staging modalities within the same procedural session.^2,3^ The combination of guided bronchoscopy, radial endobronchial ultrasound (r-EBUS), and staging linear EBUS is well established as a common procedure for the diagnosis and staging of lung cancer.^4^ Increasing lung nodule detection combined with technological advances in bronchoscopy may lead to an increase in advanced diagnostic bronchoscopy procedural volume, with a subsequent increased demand for more procedural block time.^5,6^ Even with the increasing demand, pulmonary procedure rooms still need to accommodate other bronchoscopy and pleural procedures. Thus, understanding institutional procedural time requirements is important to manage appropriate scheduling.

The currently available robotic-assisted bronchoscopy (RAB) systems allow access to lung lesions utilizing planning software, navigation, direct visualization, advanced articulation of catheters, and stability. The Ion endoluminal system (Intuitive Surgical, Sunnyvale, CA, USA) utilizes shape sensing technology for navigation, while the Monarch Platform (Ethicon, Johnson & Johnson Surgical Technologies, Raritan, NJ, USA) and Galaxy System (Noah Medical, San Carlos, CA, USA) use electromagnetic navigation. RAB systems are often used with ancillary imaging modalities including r-EBUS, fluoroscopy, and advanced 3-dimensional imaging via digital tomosynthesis, mobile or fixed cone beam computed tomography (CBCT). These technologies assist with lesion localization after navigation and confirm tools in lesion.^7^ A recent meta-analysis showed a pooled diagnostic yield of 84.3% when evaluating RAB procedures from 20 studies that sampled 1779 lesions (CI 81%–87.2%). Overall rates of pneumothorax, need for tube thoracostomy, and significant hemorrhage were 2.3%, 1.2%, and 0.5%, respectively.^8^

Current literature focuses on safety and diagnostic outcomes of robotic-assisted navigational bronchoscopy technologies with ancillary equipment, often during a facility’s initial adoption phase.^9–11^ Here we describe our institution’s current workflow for RAB biopsy procedures, detailing case times longitudinally, and including the use of other diagnostic and staging procedures.

## Methods

This was an observational study of all consecutive patients who underwent RAB procedures using the Ion endoluminal system between December 1, 2020 and October 16, 2023 to sample pulmonary lesions in a single procedure room at Clements University Hospital – UT Southwestern Medical Center (UTSW). Patients who did not have an RAB biopsy procedure were excluded. No sample size calculation was performed. The study was approved by the UTSW Institutional Review Board (STU-2021-0346).

Clinical data collected included patient demographics, lesion characteristics, and procedural specifics. The lesion location (central, middle, or peripheral) was determined using the concentric line pattern.^12^ Radial EBUS was documented as concentric, eccentric, or could not be visualized as per the procedure report and captured images. The intraprocedural use of CBCT was documented and confirmation of tool in lesion was not required. The detail of the procedure note was according to the preference and style of individual bronchoscopists with no specific template requirement. REDCap (Research Electronic Data Capture) tools at UTSW were used to collect data (supported by CTSA UL1 TR003163 grant from the National Center for Advancing Translational Science (NCATS), a component of the National Institutes of Health (NIH)).^13,14^

### Workflow

All RAB procedures at UTSW during this time were performed by Interventional Pulmonology (IP) faculty physicians. Patients were referred for targeted lung biopsies for a variety of indications including lesions suspicious for lung cancer, lung lesions for patients with pre-existing cancer to diagnose metastatic disease or obtain additional tissue per oncology request, or for non-malignant processes. Patients were either evaluated in the IP clinic or referred directly for RAB procedures after chart review by IP faculty. Patients were scheduled for a RAB with CBCT and EBUS procedure during IP block time with the bronchoscopist who had evaluated or reviewed the patient’s chart. All procedures were performed in one dedicated pulmonary procedure room with a fixed CBCT (Philips Allura FD20 with XperGuide software, Best, The Netherlands) located at Clements University Hospital. No procedures were performed in an operating room.

During this time, anesthesia support for procedures was allocated at 540 minutes per day for five days per week. RAB procedures were initially scheduled for 150–180 min, particularly during the inaugural five RAB procedures for each bronchoscopist. Based on continuous assessment and comfort of the bronchoscopist, this was subsequently reduced to 90–120 minutes.

Patients underwent same-day CT chest imaging immediately after arrival to the hospital. Our procedural technique has previously been described.^11,15^ Linear EBUS was performed as per guidelines for lung cancer staging if indicated.^16^

### Outcomes

The primary outcomes for this specific analysis are procedural times including total time the patient is in the procedure room, total time in the procedure room excluding the bronchoscopy portion, total bronchoscopy time from first scope in to last scope out, and RAB-specific bronchoscopy time. Secondary outcomes are patient demographics, nodule specific, and overall procedure characteristics. The goal of this analysis is to better understand procedural times to incorporate this information into IP procedure scheduling and block time allocation.

### Statistical analysis

Descriptive statistics are reported as counts and percentages, and medians with interquartile ranges. Diagnostic yields are calculated based on pathology and cytology results at index bronchoscopy with no additional follow-up information as described by Vachani et al.^17^ A Mann–Whitney *U* test was run to determine if there were differences between procedures that utilized ROSE as compared to no ROSE, CBCT as compared to no CBCT, or if EBUS-TBNA was performed as compared to not performed. A Kruskal–Wallis *H* test was run to determine if there were differences between the number of nodules biopsied per procedure and procedure time between various study periods. All statistical tests were two-tailed and a *p*-value of <0.05 was considered statistically significant. The data was analyzed with IBM SPSS Statistics (Version 29, IBM Corporation, Armonk, NY, USA). We present this article in accordance with the STROBE reporting checklist (Supplemental Material).^18^

## Results

Patient demographics and clinical characteristics are described in Table 1. Lesion characteristics are described in Table 2. Seven hundred RAB biopsy procedures to evaluate 776 pulmonary lesions were performed from December 1, 2020 to October 16, 2023. Procedures were divided into quartiles over time for analysis. Excluding weekends and public holidays, this resulted in 694 potential procedure days with an overall average of one RAB procedure per available day. There was an increase in the volume of RAB procedures over time (0.7 procedures per working day in period 1 compared to 1.5 procedures per working day in period 4). The increase in procedural volume over successive periods was related to the growth of our IP and RAB procedure programs. Overall, there was an increase in the number of procedures that sampled more than one lesion from nine procedures in period 1 to 25 procedures in period 4 (Table 3).

**Table 1.** Patient demographics, *n* = 700.

Median age, years	70 (62–76)
Sex	
Female	375 (53.6%)
Male	325 (46.4%)
Median body mass index	26.5 (23, 30)
Current or former tobacco smoker	447 (63.9%)
Patients with at least one medical comorbidity	556 (79.4%)
Coronary artery disease	130 (18.6%)
Chronic heart failure	67 (9.6%)
Chronic obstructive lung disease	217 (31%)
Hypertension	433 (61.9%)
Interstitial lung disease	28 (4%)
Personal history of non-lung cancer	286 (40.9%)
Personal history of lung cancer	78 (11.1%)
Transplant patient	31 (4.4%)
ASA score at time of procedure	
1	3 (0.4%)
2	132 (17.2%)
3	588 (76.5%)
4	46 (6%)
Available pre-procedure PFT (Pulmonary Function Test)	529 (76%)
Median FEV1% (Forced expiratory volume in one second)	85 (68–100)
Median FEV1/FVC% (Forced expiratory volume in one second / forced vital capacity)	72 (63–79)
Median DLCO % (Diffusion capacity of the lungs for carbon monoxide)	73 (57–87)
Available pre-procedure transthoracic echocardiogram	288 (41.6%)
Median LVEF % (Left ventricular ejection fraction)	60 (55–64.5)
RV dilation	24 (8.4%)
Elevated PAP (Pulmonary artery pressure)	47 (16.3%)

Results are presented as *n* (%) or *n* (IQR) as appropriate.

**Table 2.** Lesion characteristics, *n* = 776.

Reason lesion identified	
Incidental	273 (35.2%)
Lung cancer screening	99 (12.8%)
Cancer staging/surveillance	230 (29.6%)
Evaluating pulmonary symptoms	174 (22.4%)
Median largest lesion size dimension, mm	19 (13.5–27.8)
Location	
Central	62 (8%)
Middle	325 (42%)
Peripheral	387 (50%)
Lobe	
Right upper	245 (31.6%)
Right middle	62 (8%)
Right lower	147 (18.9%)
Left upper	213 (27.4%)
Left lower	107 (13.8%)
Mediastinal	2 (0.3%)
Lesion characteristic	
Solid	649 (83.6%)
Ground glass opacity	43 (5.5%)
Mixed	84 (10.8%)
Bronchus sign	
Present	456 (58.8%)
Absent	230 (41.2%)
Spiculation	289 (37.2%)
Median distance to pleura, mm	12 (0–26)

Results are presented as *n* (%) or *n* (IQR) as appropriate.

**Table 3.** Procedure characteristics by period and overall.

Characteristics	Period 1	Period 2	Period 3	Period 4	Overall
Procedures	175	175	176	174	700
Total days	402	263	209	176	1050
Available procedure days (excluding weekends, holidays)	265	175	134	120	694
Average procedures per work day	0.7	1	1.3	1.5	1
Nodules sampled per procedure					
1	166	156	154	149	625
2	9	17	22	24	72
3	0	2	0	1	3
Median total time in procedure room, min	105 (91.5–121.5)	109 (89–130)	92 (79–113)	86 (76–100.8)	97 (82–118.3)
Median time in room excluding bronchoscopy, min	38 (33–43)	39 (35–45)	36.5 (33–41)	36 (32–41)	38 (33–43)
Median bronchoscopy time, min	65 (53–81.5)	68 (49–89.5)	54 (42–72)	47 (39–59)	58 (44–78)
Median RAB time, min (IQR); cases data available	52 (40–74.5); 35	45 (34.5–65); 171	38 (30.8–52); 172	36 (27–47); 168	40 (31–54); 546
CBCT used during procedure	174 (99.4%)	155 (88.6%)	155 (88.1%)	151 (86.8%)	635 (90.7%)
ROSE used during procedure	170 (97.1%)	169 (96.6%)	134 (76.1%)	162 (93.1%)	635 (90.7%)
Linear EBUS-TBNA performed	61 (34.9%)	73 (41.7%)	67 (38.1%)	55 (31.6%)	256 (36.6%)
Median Linear EBUS-TBNA targets (IQR)	2 (1–2)	2 (1–3)	2 (1–3)	2 (1–3)	2 (1–3)
Pneumothorax	3 (1.7%)	5 (2.9%)	3 (1.7%)	3 (1.7%)	14 (2%)

Results are presented as *n* (%) or *n* (IQR) as appropriate.

CBCT, cone beam computed tomography; EBUS-TBNA, endobronchial ultrasound transbronchial needle aspiration; RAB, robotic-assisted bronchoscopy.

The median procedure times including total time in the procedure room, time excluding the bronchoscopy portion of the procedure, and specific bronchoscopy time remained stable in periods 1 and 2. Bronchoscopists and supporting staff were onboarding during these periods. Subsequently, overall total procedure and bronchoscopy-specific times decreased in periods 3 and 4. There was a statistically significant decrease in specific times from period 1 to 4 for median total time in procedure room (105–86 min, *p* < 0.001), median bronchoscopy time (65–47 min, *p* < 0.001), and median robotic bronchoscopy time (52–36 min, *p* < 0.001). The reductions in period 4 intraprocedural times are likely a result of bronchoscopist’s learning curve with increased experience and comfort with the technologies combined with increased volumes. Notably, the median time in the procedure room excluding the bronchoscopy portion remained stable at 38 min in period 1 and 36 min in period 4 with no significant difference (*p* = 0.131). This is likely the result of fixed time necessary for tasks during the non-bronchoscopy portion.

Overall, the median total time in the procedure room was 97 min (IQR 82–118.3) and median total bronchoscopy time was 58 min (IQR 44–78). Robotic bronchoscopy procedure times were only available in 546 procedures as this time was manually recorded on a datasheet and not entered into the electronic medical record. Due to usage of medians, the total procedure time does not equal the time in room excluding bronchoscopy when the bronchoscopy time is added. In terms of complications, there were 14 (2%) pneumothoraces during the procedures with 6 (0.9%) requiring chest tube placement (Table 3).

The usage of CBCT decreased from period 1 (99.4%) to period 4 (86.8%) with overall usage of CBCT being 90.7%. The use of ROSE was stable in periods 1, 2, and 4 ranging from 93.1% to 97.1% utilization with a notable decrease to 76.1% during period 3 which is attributed to bronchoscopist preference. Linear EBUS-TBNA was performed in 36.6% of all cases primarily for staging purposes as per guidelines,^16^ with a median of two targets sampled (IQR 1–3; Table 3).

Table 4 describes nodule and biopsy characteristics. The median lesion size for the entire study time was 19 mm (IQR 13.5–27.8). Radial EBUS was used to evaluate 99.1% of lesions, with only 10% unable to be identified in concentric or eccentric views. Biopsy tool usage was notable for high utilization of needles (96.1%) and forceps (92.1%). We used cryoprobes in periods 3–4, with overall low usage comparatively. Diagnosis at index bronchoscopy is described with number of lesions with a malignant, specific benign, non-specific benign, or non-diagnostic result.

**Table 4.** Nodule and biopsy characteristics by period and overall.

Characteristics	Period 1	Period 2	Period 3	Period 4	Overall
Nodules	183	196	196	201	776
Median lesion size largest dimension, mm	19 (13.5–28)	18.2 (13–25.6)	18.9 (13.6–27.9)	19 (13.8–27)	19 (13.5–27.8)
r-EBUS used	183 (100%)	195 (99.5%)	194 (99%)	197 (98%)	769 (99.1%)
Concentric	82 (44.8%)	98 (50.3%)	108 (55.7%)	111 (56.3%)	399 9 (51.9%)
Eccentric	80 (43.7%)	78 (40%)	65 (33.5%)	70 (35.5%)	293 (38.1%)
Not visualized	21 (11.5%)	19 (9.7%)	21 (10.8%)	16 (8.1%)	77 (10%)
Tools used					
Needle	176 (96.2%)	195 (99.5%)	186 (94.9%)	189 (94%)	746 (96.1%)
Forceps	162 (88.5%)	168 (85.7%)	189 (96.4%)	196 (97.5%)	715 (92.1%)
Brush	15 (8.2%)	16 (8.2%)	7 (3.6%)	9 (4.5%)	47 (6.1%)
Cryoprobe	0	0	10 (5.1%)	28 (13.9%)	38 (5%)
Diagnosis at index bronchoscopy, intermediate	89.6%	81.6%	86.7%	80.6%	84.5%
Malignant	103 (56.3%)	97 (49.5%)	99 (50.5%)	104 (51.7%)	403 (51.9%)
Specific benign	43 (23.5%)	46 (23.5%)	54 (27.4%)	33 (16.4%)	176 (22.6%)
Non-specific benign	18 (9.8%)	17 (8.7%)	17 (8.6%)	25 (12.4%)	77 (10%)
Non-diagnostic	19 (10.4%)	36 (18.4%)	26 (13.2%)	39 (19.4%)	120 (15.5%)

Results are presented as *n* (%) or *n* (IQR) as appropriate.

Table 5 compares characteristics based on different procedural factors. Total time in the procedure room was increased by a median of 23 min if linear EBUS-TBNA was performed (*p* < 0.001), and increased by a median of 22 min if CBCT was used as compared to fluoroscopy only (*p* < 0.001). ROSE increased the total procedure time by a median of 13 min (*p* < 0.001). Notably, there were no significant differences in procedural times including median total time in procedure room, time excluding bronchoscopy portion, and total bronchoscopy time based on the number of lesions sampled. This is due to increased utilization of linear EBUS-TBNA with one nodule (60.1%), as compared to two nodules (23.6%) and three nodules (0%).

**Table 5.** Characteristics based on various procedural factors.

Characteristics	Number of lesions				Linear EBUS-TBNA performed			CBCT used			ROSE used		
	1	2	3	*p* Value	Yes	No	*p* Value	Yes	No	*p* Value	Yes	No	*p* Value
Procedures	625	72	3		256	444	–	635	65	–	635	65	–
EBUS-TBNA	239 (60.1%)	17 (23.6%)	0 (0%)	–	256 (100%)	0 (0%)	–	234 (36.8%)	22 (33.8%)	–	241 (38%)	15 (23.1%)	–
CBCT used	566 (90.6%)	66 (91.7%)	3 (100%)	–	234 (91.4%)	401 (90.3%)	–	635 (100%)	0 (0%)	–	573 (90.2%)	62 (95.4%)	–
ROSE used	569 (91%)	63 (87.5%)	3 (100%)	–	241 (94.1%)	394 (88.7%)	–	573 (90.2%)	62 (95.4%)	–	635 (100%)	0 (0%)	–
Nodules	625	142	9	–	273	503	–	705	71	–	701	75	–
Median total time in procedure room, min	97 (81–118)	103 (90- 124.3)	105 (101–116)	0.072	112 (97–130)	89 (78–106)	<0.001	98 (85–120)	76 (69–95)	<0.001	98 (83.5–119.5)	85 (78–103)	<0.001
Median time in room, excluding bronchoscopy, min	38 (33–42)	38 (34–44)	45 (41–46)	0.244	36 (32.8–42.3)	38 (34–43)	0.170	38 (33–43)	36 (33–41)	0.307	38 (33–43)	37 (33–41)	0.331
Median total bronchoscopy time, min	57 (44–78)	62 (53–79.5)	60 (55–75)	0.089	72.5 (60–91.3)	50 (40–63)	<0.001	60 (46–79)	41 (33–54)	<0.001	59 (45–79)	47 (39–61)	<0.001

Results are presented as *n* (%) or *n* (IQR) as appropriate.

In 219 procedures that used ROSE, CBCT, and performed linear EBUS-TBNA while sampling 233 nodules (one nodule was sampled in 205 cases, two nodules were sampled in 14 cases), the median total time in the procedure room was 114 min (IQR 98–131), median non-bronchoscopy time was 37 min (IQR 32.5–43), and median bronchoscopy time was 77 min (IQR 62–94).

## Discussion

In a single center over 34 months, 3147 pulmonary procedures were performed in one interventional pulmonary room, with an average room turnover time of 30 min between cases. Among these cases included 700 RAB procedures with an overall average of one RAB procedure per available workday with increasing volume noted over time. Procedural time including time in the room, bronchoscopy time, and RAB time decreased over the study period, while non-bronchoscopy time remained stable secondary to fixed tasks including patient transfer and positioning, induction, and emergence from anesthesia. Procedures were longer if linear EBUS-TBNA was performed if ROSE or CBCT were utilized. We surmise the overall intraprocedural efficiency improved over time secondary to increasing case volume, and provider/staff experience.

The available advanced diagnostic bronchoscopy literature focuses on outcomes including diagnostic yield and complications with minimal mention of procedural time requirements. Verma et al. described their experience with the efficiency of performing pulmonary procedures including bronchoscopy with bronchoalveolar lavage (BAL), BAL and transbronchial biopsy, linear EBUS-TBNA, and medical thoracoscopy under moderate sedation over a period of 3 months in the endoscopy unit within a teaching hospital. Mean procedure time (scope in to scope out) for all procedures was variable based on the nature of the procedure performed with a case turnaround time of 35 ± 46 min. The main causes of inefficiencies were delayed start time, increased turnover times, and underutilization of time due to inefficient scheduling, which created a backlog of patients requiring procedures.^19^ Kravac et al. performed a quality improvement project to evaluate and improve the efficiency of patient flow in the bronchoscopy unit. Among a total of 31 diagnostic, advanced diagnostic, or therapeutic procedures, the average case duration was 63 min with an average room turnover time of 30 min.^20^ A key factor identified in efficiency and overall volume in endoscopy units is turnover/turnaround time.^21^

The current shape-sensing RAB literature reports procedure times that range from 55 to 78 min.^9,10,22–24^ These times are variably calculated and reported (for example: robotic docking time to undocking time, robotic catheter insertion to removal, or not specified) without mention of other aspects of procedural time. Kalchiem-Dekel et al. noted a significant difference in median procedural time based on number of nodules sampled during the procedure.^10^

Given the complexities of workflow and the potential need to perform various procedures in the same session, there can be significant variability in the length of time necessary to successfully complete all aspects of RAB procedures. This current analysis does not consider or include the use of emerging technologies, or additional bronchoscopic procedures such as marking techniques or tumor ablation. Consideration of appropriate time requirements depending on the nature of the planned procedures is necessary. If inadequate time is allocated, then delays in subsequent cases with the potential for cancellation of procedures later in the day may occur. Alternatively, if unnecessary time is allocated to specific cases then underutilization of block time may occur, with a resulting backlog of cases. Such scheduling inefficiencies can lead to poor patient satisfaction, and poor utilization of supporting staff and services, and may potentially affect procedure outcomes.

Our study limitations include the nature of this being a single-center observational study with RAB procedures performed by IP physicians in a pulmonary procedure room. Generalizability may be limited based on specific institutional experiences, procedural volume, block time availability, staff training and familiarity with workflow, and use of advanced imaging. We did not assess outcomes based on individual providers, as the primary goal was to evaluate timing aspects at an institutional level. We recognize that individual bronchoscopists may have different practice patterns, learning curves, and outcomes for navigational bronchoscopy procedures that affect their individual utilization of procedural block times.^25^ Overall, 96% of cases were performed by four physicians at our institution, and physician caseload varied by period. There was also variability in CBCT use during procedures by physicians ranging from 83.7% to 98.2% over this time.

The primary goal of this report is to share our institutional experience with the initial 700 RAB procedures with a specific focus on the necessary time requirements and progressive change over time. During this time we did not specifically undertake any efforts to optimize procedural time by streamlining RAB to single days or have any limitations on use of CBCT for these procedures. Our institutional room turnover time for pulmonary has remained stable at 28–30 min prior to and after the introduction of RAB and CBCT. Turnover time is secondary to necessary tasks including appropriate handling of specimens and transport to lab, and room preparation for the subsequent case (both from bronchoscopy and anesthesia perspective). We continue to periodically revise our procedural scheduling and block times based on ongoing RAB procedural data collection.

## Conclusion

Understanding institutional experiences in RAB and related adjunct procedure case times may assist with optimizing case scheduling and procedural block time utilization. The concomitant use of CBCT imaging, ROSE, and linear EBUS-TBNA for staging can significantly lengthen the total RAB procedure time and should be factored in the bronchoscopy scheduling and procedural block times. Institutions performing RAB procedures should consider continuous re-evaluation of specific procedural times in order to maximize procedure and block time efficiency.

## Supplemental Material

sj-docx-1-tar-10.1177_17534666241277668 – Supplemental material for Procedural times with robotic-assisted bronchoscopy: a high volume single-center study Supplemental material, sj-docx-1-tar-10.1177_17534666241277668 for Procedural times with robotic-assisted bronchoscopy: a high volume single-center study by Kim Styrvoky, Audra Schwalk, David Pham, Kristine Madsen, Hsienchang Chiu and Muhanned Abu-Hijleh in Therapeutic Advances in Respiratory Disease

## Declarations

**Ethics approval and consent to participate:** The study was conducted in accordance with the Declaration of Helsinki (as revised in 2013). The study was approved by the UT Southwestern Institutional Review Board (STU-2021-0346) and individual consent for this analysis was waived. We are reporting a study of medical records, and the IRB waived the requirement for informed consent for inclusion in this study.

**Consent for publication:** Not applicable.

**Availability of data and materials:** Individual participant data that underlie the results reported in our article after de-identification will be shared 3 months to 5 years after this article is published with researchers whose data have been approved for use by an independent review committee for advancement of bronchoscopy. Requests may be sent to the corresponding author. Data sharing agreement will be required to be signed.
