# Supplementary material for: Procedural times with robotic-assisted bronchoscopy: a high volume single-center study
Source: Ther Adv Respir Dis. 2024 Sep 5;18:17534666241277668. doi: 10.1177/17534666241277668 (PMC11378166; doi:10.1177/17534666241277668)
Supplement: sj-docx-1-tar-10.1177_17534666241277668 – Supplemental material for Procedural times with robotic-assisted bronchoscopy: a high volume single-center study [file sj-docx-1-tar-10.1177_17534666241277668.docx]

STROBE Statement—checklist of items that should be included in reports of observational studies

| **Section/item** | **Item No** | **Recommendation** | **Reported on Page Number/Line Number** | **Reported on Section/Paragraph** |
| --- | --- | --- | --- | --- |
| **Title and abstract** | 1 | (a) Indicate the study’s design with a commonly used term in the title or the abstract | p 1, lines 3 | Title |
|  |  | (b) Provide in the abstract an informative and balanced summary of what was done and what was found | p 3, lines 36-45 | Design, methods, results |
| **Introduction** | | | | |
| Background/ rationale | 2 | Explain the scientific background and rationale for the investigation being reported | p 4, lines 51-82 | Introduction, paragraphs 1-3 |
| Objectives | 3 | State specific objectives, including any prespecified hypotheses | p 5, lines 81-82 | Introduction, paragraph 3 |
| **Methods** | | | | |
| Study design | 4 | Present key elements of study design early in the paper | p 5, lines 85-89 | Methods, paragraph 1 |
| Setting | 5 | Describe the setting, locations, and relevant dates, including periods of recruitment, exposure, follow-up, and data collection | p 5, lines 85-98 | Methods, paragraph 1-2 |
| Participants | 6 | (a) ***Cohort study***—Give the eligibility criteria, and the sources and methods of selection of participants. Describe methods of follow-up  ***Case-control study***—Give the eligibility criteria, and the sources and methods of case ascertainment and control selection. Give the rationale for the choice of cases and controls  ***Cross-sectional study***—Give the eligibility criteria, and the sources and methods of selection of participants | p 5, lines 85-98 | Methods, paragraph 1-2 |
|  |  | (b) ***Cohort study***—For matched studies, give matching criteria and number of exposed and unexposed  ***Case-control study***—For matched studies, give matching criteria and the number of controls per case | N/A - did not perform matched study | N/A- did not perform matched study |
| Variables | 7 | Clearly define all outcomes, exposures, predictors, potential confounders, and effect modifiers. Give diagnostic criteria, if applicable | p 6, lines 119-125, 128-129 | Methods, paragraphs 6-7 |
| Data sources/ measurement | 8* | For each variable of interest, give sources of data and details of methods of assessment (measurement). Describe comparability of assessment methods if there is more than one group | p 5, lines 90-98 | Methods, paragraph 2 |
| Bias | 9 | Describe any efforts to address potential sources of bias | N/A - included all patients | with available data |
| Study size | 10 | Explain how the study size was arrived at | p 5, lines 85-89 | Methods, paragraph 1 |
| Quantitative variables | 11 | Explain how quantitative variables were handled in the analyses. If applicable, describe which groupings were chosen and why | p 7, lines 127-136 | Methods, paragraph 7 |

| Statistical methods | 12 | (a) Describe all statistical methods, including those used to control for confounding | p 7, lines 127-136 | Methods, paragraph 7 |
| --- | --- | --- | --- | --- |
|  |  | (b) Describe any methods used to examine subgroups and interactions | p 7, lines 129-136 | Methods, paragraph 7 |
|  |  | (c) Explain how missing data were addressed | N/A - data available | N/A data available |
|  |  | (d) ***Cohort study***—If applicable, explain how loss to follow-up was addressed  ***Case-control study***—If applicable, explain how matching of cases and controls was addressed  ***Cross-sectional study***—If applicable, describe analytical methods taking account of sampling strategy | N/A - this paper focuses  on procedural specific data. | N/A - this paper focuses on procedural specific data |
|  |  | (e) Describe any sensitivity analyses | N/A - not performed | N/A - not performed |
| **Results** | | | | |
| Participants | 13* | (a) Report numbers of individuals at each stage of study—eg numbers potentially eligible, examined for eligibility, confirmed eligible, included in the study, completing follow-up, and analysed | p 7, line 139 | Results, paragraph 1 |
|  |  | (b) Give reasons for non-participation at each stage | N/A - this paper focuses | on procedural specific data |
|  |  | (c) Consider use of a flow diagram | N/A - all consecutive | biopsy procedures included |
| Descriptive data | 14* | (a) Give characteristics of study participants (eg demographic, clinical, social) and information on exposures and potential confounders | p 7, line 139-140 | Results, paragraph 1 |
|  |  | (b) Indicate number of participants with missing data for each variable of interest | p 8, lines 162-164 | Results, paragraph 3 |
|  |  | (c) ***Cohort study***—Summarise follow-up time (eg, average and total amount) | N/A - this paper focuses | on procedural specific data |
| Outcome data | 15* | ***Cohort study***—Report numbers of outcome events or summary measures over time | p 7, lines 139-190 | Results, paragraph 1-7 |
|  |  | ***Case-control study***—Report numbers in each exposure category, or summary measures of exposure | N/A - cohort study | N/A - cohort study |
|  |  | ***Cross-sectional study***—Report numbers of outcome events or summary measures | N/A - cohort study | N/A - cohort study |
| Main results | 16 | (a) Give unadjusted estimates and, if applicable, confounder-adjusted estimates and their precision (eg, 95% confidence interval). Make clear which confounders were adjusted for and why they were included | N/A - not performed | N/A - not performed |
|  |  | (b) Report category boundaries when continuous variables were categorized | N/A - not performed | N/A - not performed |
|  |  | (c) If relevant, consider translating estimates of relative risk into absolute risk for a meaningful time period | N/A - not performed | N/A - not performed |
| Other analyses | 17 | Report other analyses done—eg analyses of subgroups and interactions, and sensitivity analyses | N/A - not performed | N/A - not performed |
| **Discussion** | | | | |
| Key results | 18 | Summarise key results with reference to study objectives | p 9, lines 193-201 | Discussion, paragraph 1 |
| Limitations | 19 | Discuss limitations of the study, taking into account sources of potential bias or imprecision. Discuss both direction and magnitude of any potential bias | p 11, lines 230-239 | Discussion, paragraph 5 |

| Interpretation | 20 | Give a cautious overall interpretation of results considering objectives, limitations, multiplicity of analyses, results from similar studies, and other relevant evidence | p 10, lines 202-219, 11 p  240-248 | Discussion, paragraphs 2,  3, 6 |
| --- | --- | --- | --- | --- |
| Generalisability | 21 | Discuss the generalisability (external validity) of the study results | p 11, lines 231-233 | Discussion, paragraph 5 |
| **Other information** | | | | |
| Funding | 22 | Give the source of funding and the role of the funders for the present study and, if applicable, for the original study on which the present article is based | N/A - no funding source | N/A - no funding source |

*Give information separately for cases and controls in case-control studies and, if applicable, for exposed and unexposed groups in cohort and cross-sectional studies.

**Note:** An Explanation and Elaboration article discusses each checklist item and gives methodological background and published examples of transparent reporting. The STROBE checklist is best used in conjunction with this article (freely available on the Web sites of PLoS Medicine at [http://www.plosmedicine.org/,](http://www.plosmedicine.org/) Annals of Internal Medicine at [http://www.](http://www/) annals.org/, and Epidemiology at [http://www.epidem.com/).](http://www.epidem.com/)) Information on the STROBE Initiative is available at [www.strobe-statement.org.](http://www.strobe-statement.org/)

n/a
